# A cross-sectional study of male and female C57BL/6Nia mice suggests lifespan and healthspan are not necessarily correlated

**DOI:** 10.18632/aging.101059

**Published:** 2016-10-02

**Authors:** Kathleen E. Fischer, Jessica M. Hoffman, Lauren B. Sloane, Jonathan A.L. Gelfond, Vanessa Y. Soto, Arlan G. Richardson, Steven N. Austad

**Affiliations:** ^1^ Department of Biology, University of Alabama at Birmingham, Birmingham, AL 35294, USA; ^2^ The Barshop Institute for Longevity and Aging Studies, University of Texas Health Science Center, San Antonio, TX 78245, USA; ^3^ Division of Liberal Arts and Sciences at SUNY Delhi, Delhi, NY 13753, USA; ^4^ Department of Geriatric Medicine, Oklahoma University Health Science Center, Oklahoma City VA Medical Center, Oklahoma City, OK 73104, USA

**Keywords:** healthspan, lifespan, physiological function, aging, longevity, sex differences

## Abstract

Lifespan provides a discrete metric that is intuitively appealing and the assumption has been that healthspan is extended concomitant with lifespan. Medicine has been more successful at extending life than preserving health during aging. Interventions that extend lifespan in model organisms do not always result in a corresponding increase in healthspan, suggesting that lifespan and healthspan may be uncoupled. To understand how interventions that extend life affect healthspan, we need measures that distinguish between young and old animals. Here we measured age-related changes in healthspan in male and female C57BL/6JNia mice assessed at 4 distinct ages (4 months, 20 months, 28 months and 32 months). Correlations between health parameters and age varied. Some parameters show consistent patterns with age across studies and in both sexes, others changed in one sex only and others showed no significant differences in mice of different ages. Few correlations existed among health assays, suggesting that physiological function in domains we assessed change independently in aging mice. With one exception, health parameters were not significantly associated with an increased probability of premature death. Our results show the need for more robust measures of murine health and suggest a potential disconnect between health and lifespan in mice.

## INTRODUCTION

Interventions that extend longevity in model laboratory organisms have proliferated at least since McCay et al [1] published their first study on dietary restriction in 1935. Traditionally, such interventions have been assumed to retard aging itself based on their ability to increase mean and maximum lifespan. The emphasis on longevity metrics alone made some sense in that longevity seemingly provides an unambiguous endpoint that has been assumed to be necessarily correlated with a general age-related physiological decline. While this may often be the case, it is not necessarily so. Indeed, human females live longer lives than males, but also suffer greater age-related morbidity by a number of measures [2]. In laboratory species, long-lived worm genotypes are often outcompeted by shorter-lived genotypes [3, 4] and some evidence suggests that by a number of measures *some* long-lived worm genotypes are less healthy than the standard N2 genotype even relatively early in life [5]. As a major goal of basic aging research is to develop interventions that will enhance and prolong health in humans, it would be beneficial to the field to determine for all model organisms, which interventions extend health and which extend only life.

Health assessment even in humans is not straightforward because the definition of health varies depending on who is doing the assessment and why. Most people would define themselves as healthy if they are capable of doing the things that they wish to do. In this sense, self-rated health is a valuable – perhaps the most valuable – health metric we have. Demographers of public health may distinguish between health, morbidity, and disability [6], and geriatricians may categorize the elderly into other classifications depending on what diseases they have, how well those are controlled, their sense of psychological well-being, how functionally independent they are, or how likely to recover from an acute health challenge such as a fall or fracture [7-10]. Such assessments may be used to develop treatment plans, for screening, or for prognosis.

In animal studies, many human metrics noted above simply cannot be reverse-translated. Issues such as degree of functional independence and psychological well-being are difficult at best, meaningless at worst, to implement in animal models. Most effort in animal studies has gone into developing either “biomarkers of aging,” defined variously by various investigators, but mostly focusing on indicators of future mortality [11-13], or construction of frailty indices to serve as indicators of future adverse health outcomes.

Because efforts to develop a cognate battery of tests to assess healthspan in mice and other model organisms have met with varying degrees of success [9, 14-20] we have taken a different approach. We feel that important indicators of health that can be commonly addressed in humans and in mice can be roughly categorized as those associated with age-related decreasing strength and mobility and those associated with decreasing cognitive capabilities. To this end, we present here an analysis of age-related change in commonly measured, noninvasive parameters associated with age-related changes in energetics, strength and mobility in the commonly used C57BL/6 mouse strain, and we determined to what extent these health parameters were associated with premature death. Our study is distinguished from other similar studies [18, 21] in that we performed all our tests the same way in both sexes and also that we performed tests on animals at the advanced age of 32+ months. The importance of this latter point is that whereas clinical geriatricians are often treating patients considerably above the age of mean survival for the population, mouse studies have typically avoided such elderly mice. However, if results are to be compared between mice and humans such ages necessarily must be assessed.

## RESULTS

### Body composition

**Figure 1:**
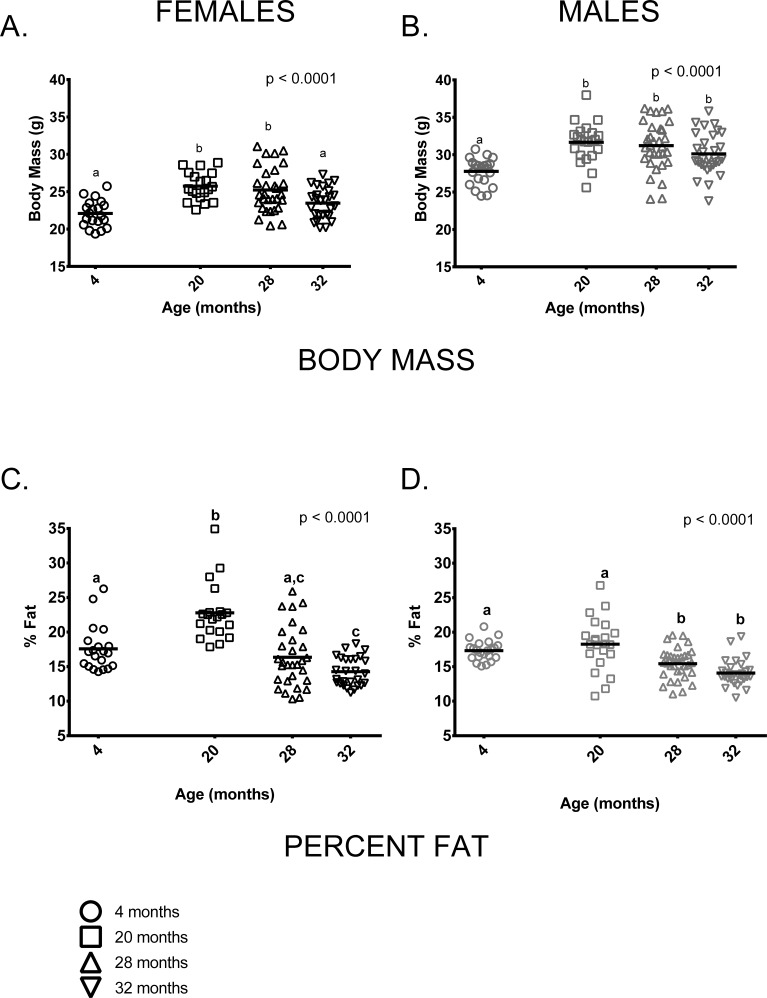
Body size and composition change with age in female and male mice (**A**) 4-month-old females were smaller than 20 and 28-month-old females (p < 0.0001 in both cases) but similar to 32-month-old females (p = 2.276). (**B**) 4-month -old males were smaller than 20, 28 and 32-month-old male mice (p <0.0001, p <0.0001and p = 0.018, respectively). (**C**) 20-month-old females had a greater percentage of body fat than 4, 28 and 32-month-old female mice (p < 0.0001 in all cases). 4-month -old females had a greater proportion of body fat than 32-month-old females (p = 0.014) but did not differ from 28-month-old females. (**D**) 4-old males had a greater proportion of fat than either 28 or 32-month-old males (p = 0.036, p < 0.0001, respectively), as did 20-month-old males (p = 0.0003 and p < 0.0001). But 4 and 20-month-old males and 28 and 32-month-old males did not differ (p > 0.999, p = 0.179, respectively). Post-hoc tests subject to Bonferroni correction for multiple comparisons. Body composition sample size: Females n = 20, 20, 30 and 27 for 4, 20, 28 and 32 months; Males n = 22, 22, 32 and 30 for 4, 20, 28 and 32 months.

As is typically seen in C57BL/6 mice, mean body mass of males was greater than females at all ages measured. Somewhat surprisingly, there was no weight difference between the 20 and 28-month age groups in either males or females ($\bar{X\;}$ difference < 0.5g in both sexes). This is distinct from previous studies of C57BL/6 (e.g. [19, 22, 23]), which show relatively consistent weight gain up to about 15-20 months of age and a decline thereafter. 32-month-old females weighed significantly less than females in the 20 or 28-month-old age groups and males showed a similar, albeit non-significant, trend in the same direction. On average, 4-month-old female mice weighed less than 20 and 28-month-old females but were similar to 32-month-old females (Figure 1A). 4-month-old male mice weighed less than older males but body mass did not differ among 20, 28 and 32-month-old males (Figure 1B). Body composition also differed among the four age groups. 4-month-old males and females both had less fat-free mass compared to older mice of all ages (p < 0.0001 in both cases, data not shown). The proportion of body fat differed among the age groups in an age by sex-specific fashion (age * sex interaction, p = 0.002). Among females, percent body fat was greatest in the 20-month-old cohort; 4-month-old females did not differ from 28-month-old females in percent body fat, but did differ from 32-month-olds (Figure 1C). Males in the 4 and 20-month-old age groups had a similar proportion of body fat and had a higher proportion of body fat than males in the 28 and 32-month-old age groups (Figure 1D). Females in the 20-month-old age group were proportionately fatter than males in all age groups (p < 0.0001 in all cases). Males and females in the 4-month-old group had similar percent body fat and did not differ from 20-month old males or 28-month-old animals of either sex. Males and females in the two oldest age groups (28 and 32 months) had similar percent body fat.

### Activity and energetics

**Figure 2:**
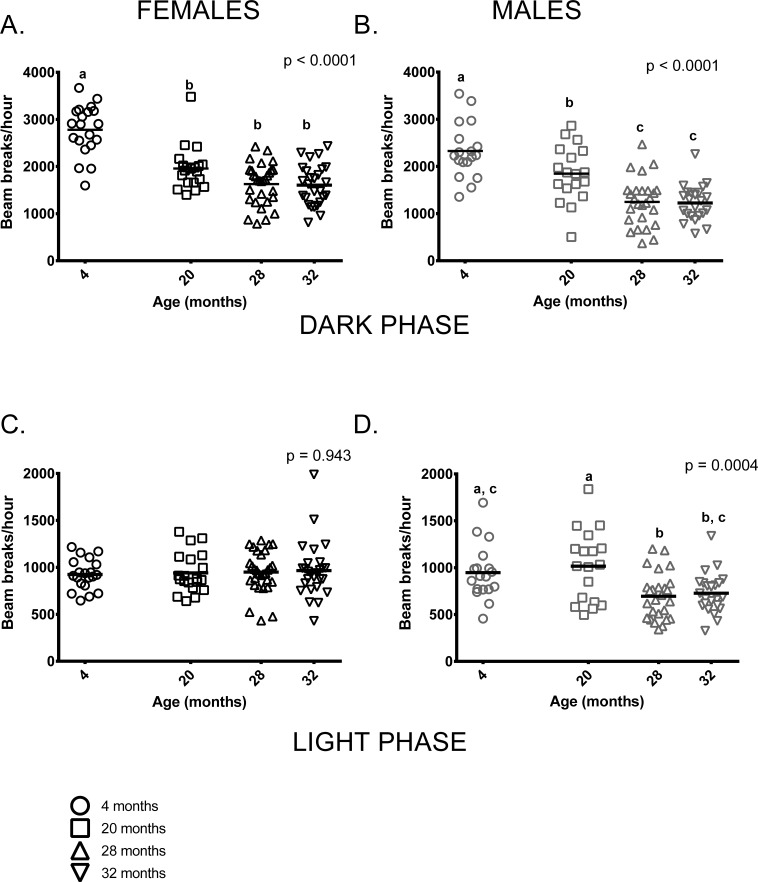
Total activity levels were lower in older mice during the dark phase; activity levels of males, but not females differed among age groups during the light phase of the 24-hour light-dark cycle (**A**) 4-month -old female mice were more active during the dark (=active) phase than females in all other age groups (p < 0.0001 in all cases). 20-month-old females did not differ from 28 and 32-month-old females (p = 0.088, p = 0.066, respectively) and 28 and 32-month-old females did not differ from each other (p > 0.999) in dark phase activity. (**B**) 4-month -old males were more active than 20, 28 and 32-month-old males during the dark phase (p = 0.039, p < 0.0001 and p < 0.0001, respectively). 20-month-old males were more active than 28 and 32-month-old males (p = 0.002 in both cases). (**C**) Female activity levels during the light phase do not differ between the four age groups. (**D**) Male activity during light phase was highly variable in all age groups. 4-month -old males' activity levels did not differ from 20 and 32-month old males (p > 0.999 and p = 0.088, respectively). 4-month -olds were more active than 28-month -olds (p = 0.03) and 20-month-olds were more active than both 28 and 32-month-old males (p = 0.002 and p = 0.010, respectively). Post-hoc tests subject to Bonferroni correction for multiple comparisons. Females n = 20, 20, 30 and 27 for 4, 20, 28 and 32 months. Males n = 18, 18, 26 and 23 for 4, 20, 28 and 32 months.

Both age and sex influenced activity levels in these mice during the dark (=active) phase. Activity levels during the dark phase were reduced in older mice and females were more active than males at all ages (p < 0.0001 in both cases). Four month-old females were more active than older females, who had similar activity levels regardless of age. Similarly, 4-month-old males were more active than older males but 20-month-old males were more active than 28 and 32-month-old mice (Figure 2A,B). Male, but not female, activity during the light phase differed between the age groups; however, individual variability in males' light phase activity was high. (Figure 2C,D).

**Figure 3:**
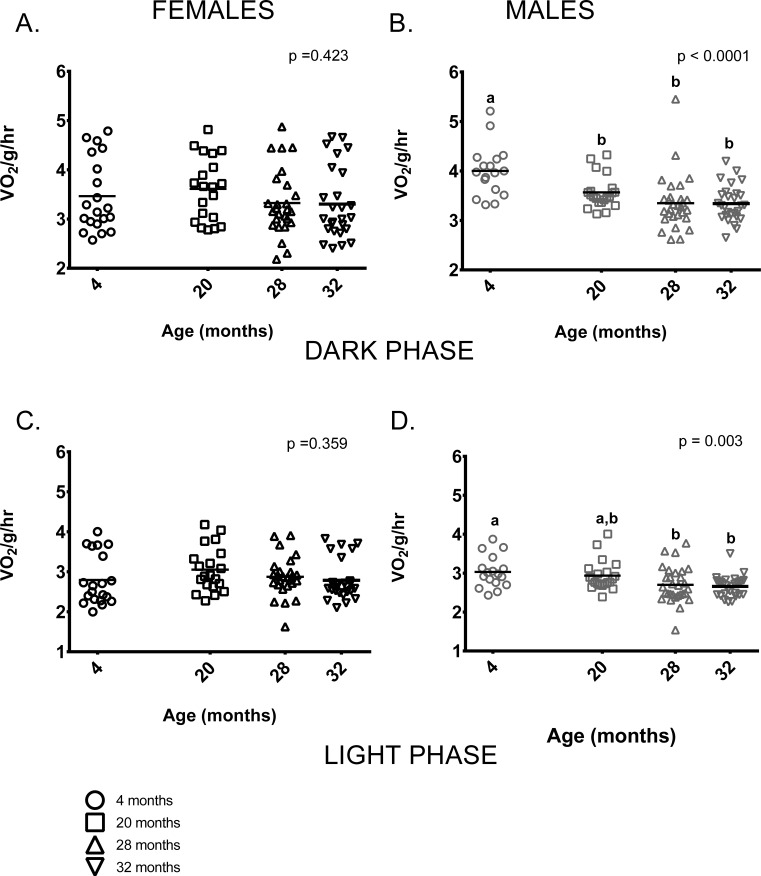
Metabolic rate differed between the age groups in male, but not female mice (**A**) Female mice of all ages had similar metabolic rates during the dark (=active) phase. (**B**) During the dark phase, 4-month -old male mice had higher metabolic rates on average than 20, 28 and 32-month-old males (p = 0.025, p < 0.0001, p < 0.0001 respectively). 20-month-old males also had higher mass-specific metabolic rates compared to 28 and 32-month-olds (p = 0.002 in both cases). 28 and 32- month-old males did not differ (p > 0.999). (**C**) Female mice of all ages had similar metabolic rates during the light (=inactive) phase (p = 0.943). (**D**) During the light phase, 4-month -old male mice had higher metabolic rates on average than 28 or 32-month-old males (p = 0.033, p = 0.012, respectively), as did 20-month-old males (p = 0.002, p = 0.010, respectively). 28 and 32- month-old males did not differ from one another (p > 0.999). Post-hoc tests subject to Bonferroni correction for multiple comparisons. Metabolism sample size: Females n = 20, 20, 26 and 26 for 4, 20, 28 and 32 months; Males n = 17, 21, 29 and 28 for 4, 20, 28 and 32 months.

Female mice showed no age-related differences in mass-specific metabolic rate during the dark (=active) or during light (=inactive) phases (Figures 3A,C). In contrast, 4-month-old males had higher mass-specific metabolic rates during the dark phase than males in all other age groups and higher rates than 28 and 32-month-old males during the light phase. 20-month-old males had higher mass-specific metabolic rates compared to 28 and 32-month-old males during both light and dark phases (Figure 3B,D).

**Figure 4:**
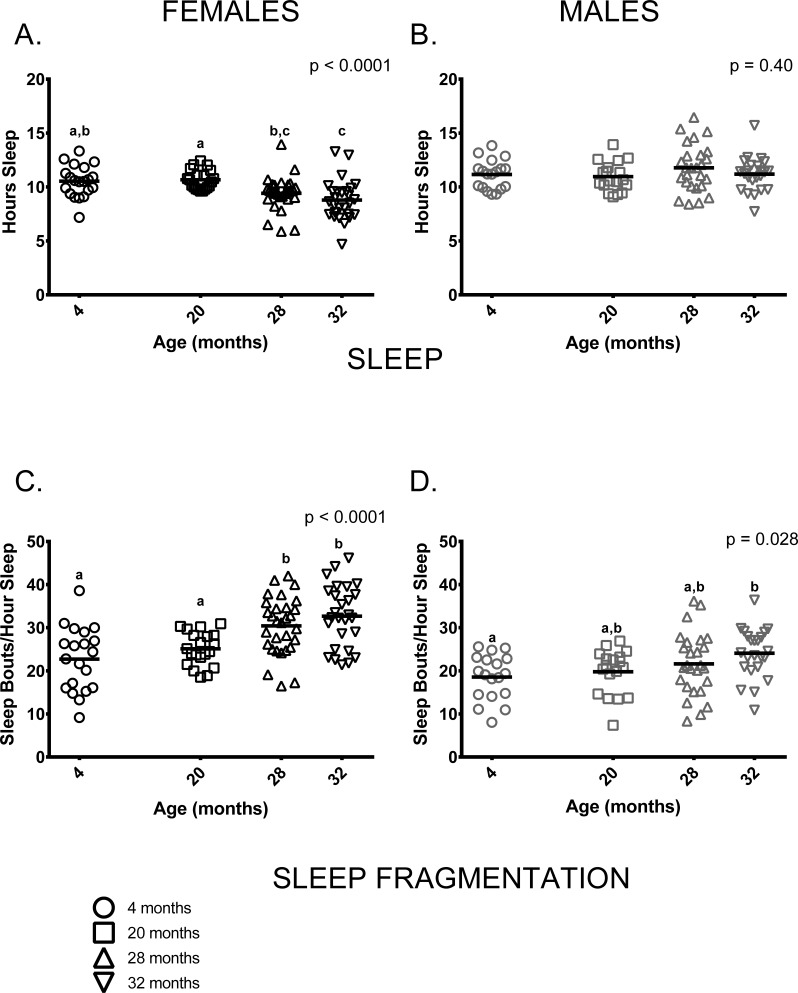
Sleep fragmentation was greater in older mice and older females slept less compared to younger ones (**A**) On average, older female mice (ages 28 and 32 months) slept less than 20-month-old females (p = 0.029, p = 0.0003, respectively) and 32-month-oldfemales slept less than 4-month -old females (p = 0.001). 4-month -old females did not differ from 20-month-old females or 28-month -old females (p >0.999, p = 0.068, respectively), and 28-month -old females did not differ from 32-month-olds (p = 0.796) in the amount of time spent sleeping. (**B**) Male mice did not show age differences in total amount of sleep measured over a 24-hour period (p = 0.40). (**C**) Sleep fragmentation was greater in older females (ages 28 and 32 months) than in 4-month -old females (p = 0.0006, p < 0.0001, respectively) and 20-month-old females (p = 0.036, p = 0.001, respectively). Sleep fragmentation did not differ between 28 and 32-month-old (p >0 0.999). (**D**) Sleep fragmentation was greater in older male mice (p = 0.028); however, in pair-wise comparisons only 4-month and 32-month-old males were significantly different (p = 0.030). Post-hoc tests subject to Bonferroni correction for multiple comparisons. Females n = 20, 20, 30 and 27 for 4, 20, 28 and 32 months. Males n = 18, 18, 26 and 23 for 4, 20, 28 and 32 months.

Similar to previous studies on C57BL/6 by our group [23, 24], sleep and sleep fragmentation at various ages differed in a sex-specific fashion (sleep, age * sex p = 0.001; sleep fragmentation, age * sex p < 0.0001). Female, but not male mice, showed age-related differences in total time spent sleeping (Figure 4A,B). Older female mice (ages 28 and 32 months) slept less than 20-month-old females, 4-month-old females slept more than 32-month-old females and 28-month-old females did not differ from 32-month-olds (Figure 4A). In contrast to females, total amount of time males spent sleeping did not differ between age groups (Figure 4B). Male and female mice also showed distinct age-associated patterns of sleep fragmentation as measured during the light (=inactive) phase. Sleep fragmentation was greater in older females (ages 28 and 32 months) than in younger ones (4 and 20 months) (Figure 4C). Overall, sleep fragmentation was greater in older males but the differences only reached significance in pair-wise comparisons of 4 and 32-month-old males (Figure 4D).

A hallmark of human aging is the decline of strength, balance, coordination and neuromuscular function. In humans, measures of gait, balance, coordination and grip strength are commonly used to predict future age-related morbidity and mortality [7, 25-28]. We used a cognate set of assays here that are commonly used to measure age-related changes in strength and motor function in mice.

#### Gait

**Figure 5:**
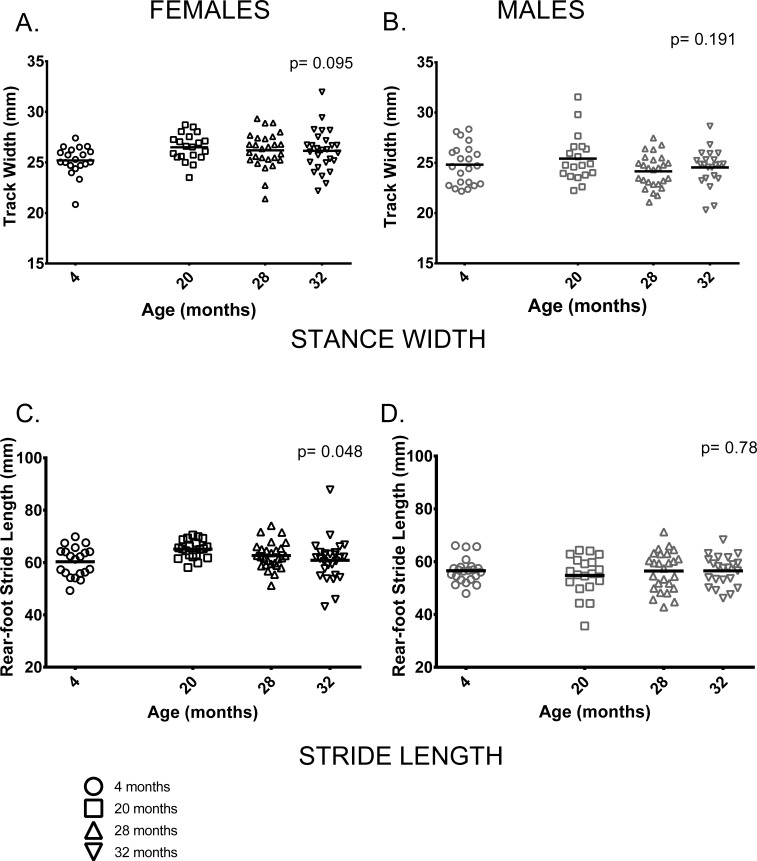
Female, but not male, stride length differed among age groups; gait width showed no differences among age groups **(A, B)** Gait width did not differ among the four age groups in female (p = 0.095) or male (p = 0.191) mice. **(C)** Mean rear-foot stride length among females in the four age groups was marginally different (p = 0.048). **(D)** Rear-foot stride length did not differ between males in the 4 age groups (p = 0.786). Post-hoc tests subject to Bonferroni correction for multiple comparisons. Sample size: Females n = 20, 20, 27 and 27 for 4, 20, 28 and 32 months; Males n = 22, 19, 26 and 22 for 4, 20, 28 and 32 months.

We measured a range of gait parameters and chose to use two parameters of gait, rear foot stride length and rear foot stance width based on their use in previous studies and because tests of neuromuscular function performed on these animals were done on the rear limbs and tail [29]. In contrast to previous studies by our group and others [19, 20, 21, 23, 24, 30] two parameters of gait, stride length and gait width, did not differ between age groups or only differed marginally. Stance width, a measure of the distance between left and right hind feet, did not differ between age groups in either sex (Figure 5A,B). Similarly, rear-foot stride length did not differ between male mice of different ages and showed only a marginally significant difference between age groups in females (p = 0.048) (Figure 5C,D).

#### Rotarod

**Figure 6:**
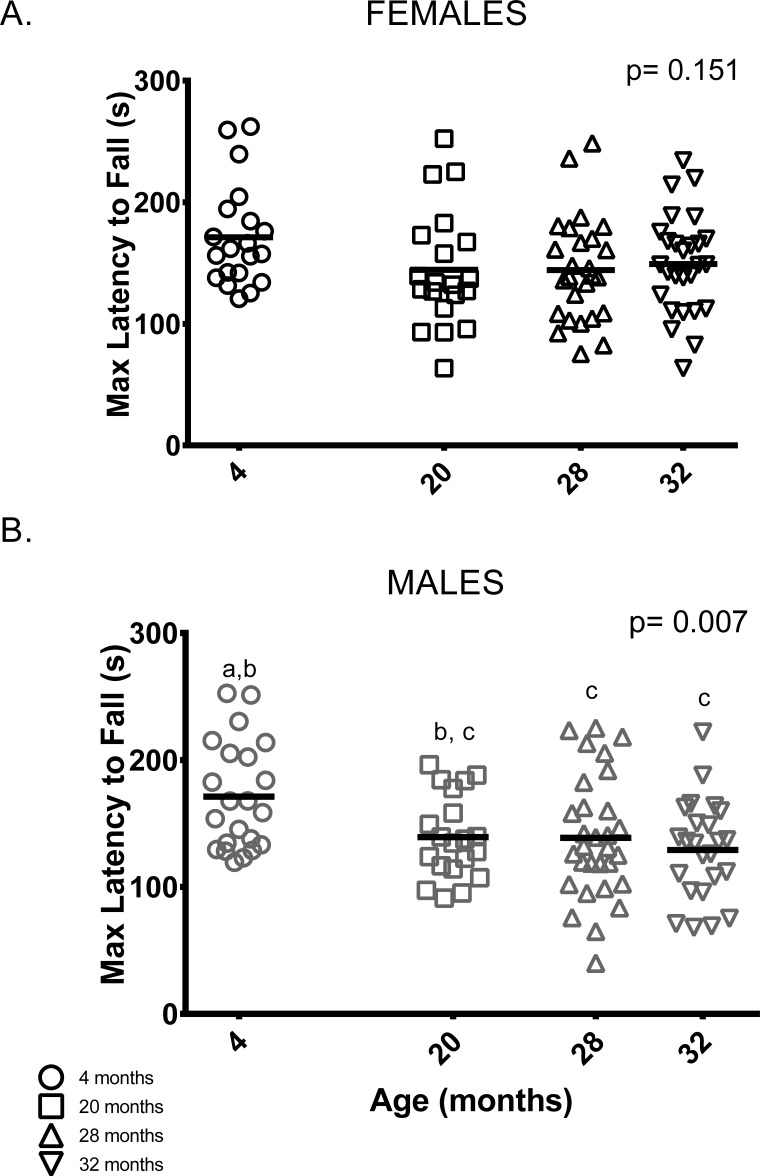
Younger males, but not females, performed better than older mice on the rotarod test (**A**) Rotarod performance varied among female mice of all age groups. (**B**) 4-month -old males stayed on the rotarod longer than 28 and 32-month -old males (p = 0.042 and p = 0.006, respectively). Post-hoc tests subject to Bonferroni correction for multiple comparisons. Rotarod sample size: Females n = 20, 20, 26 and 27 for 4, 20, 28 and 32 months; Males n = 22, 20, 30 and 24 for 4, 20, 28 and 32 months.

Unlike previous studies [21, 23, 24], female rotarod performance (maximum latency to fall out of six trials) did not differ between age groups (Figure 6A). On the other hand, male rotarod performance was better in younger animals: 4-month-old males remained on the rotarod longer than 28 and 32-month-old male mice. Rotarod performance by 20-month-old males was indistinguishable from all other age groups (Figure 6B).

#### Grip

**Figure 7:**
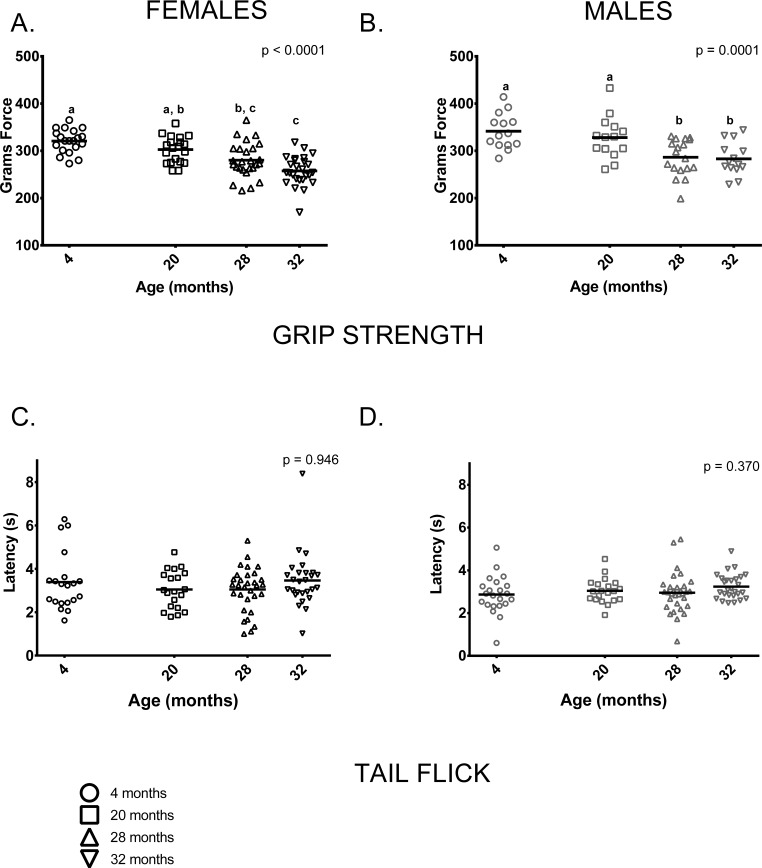
Grip strength was reduced in older mice compared to younger mice; performance on the tail flick test did not differ between age groups **(A)** Female grip strength was reduced in older females compared to younger ones (p < 0.0001). Grip strength was greater in 4-month -old females than 28 and 32-month -olds (p = 0.0002 and p< 0.0001, respectively) and did not differ from 20-month-olds (p =0.431). Grip strength measured in 20-month-old females was greater than that of 32-month -olds (p < 0.0001), but not 28-month -olds (p = 0.0942). **(B)** Grip strength was similar in 4 and 20-month old males (p > 0.999); both had greater grip strength than 28-month -old (p = 0.001 and p = 0.024, respectively) and 32-month -old males (p = 0.001 and p = 0.021, respectively). Grip strength was similar in 28 and 32-month old males (p > 0.999) **(C, D)** Performance on the tail flick test did not differ between age groups in female (p = 0.422) or male (p = 0.370) mice. Post-hoc tests subject to Bonferroni correction for multiple comparisons. Grip strength sample size: Females n = 20, 20, 31 and 27 for 4,20, 28 and 32 months; Males n = 14, 14, 18 and 14 for 4, 20, 28 and 32 months. Tail flick sample size: Females n = 20, 20, 26and 26 for 4, 20, 28 and 32 months; Males n = 22, 20, 31 and 27 for 4, 20, 28 and 32 months.

Grip strength was reduced in older mice (28 and 32-month-olds) compared to 4-month-olds; mean grip strength was 20% lower in 32-month-old females and 16% lower in 32-month-old males than in sex-matched 4-month-olds. Males had greater grip strength than age-matched females at all ages measured (p < 0.0001 in all cases). 4-month-old and 20-month-old females' grip strength did not differ and both were stronger than 32-month-olds, but only 4-month-olds were stronger than 28-month-old females. (Figure 7A). Younger male mice (ages 4 and 20 months) had comparable grip strength and were stronger when compared to older mice (ages 28 and 32) (Figure 7B).

The tail flick test is a commonly used indirect measure of peripheral sensation; we performed this test to determine whether it could pick up differences as measured by the more invasive nerve conduction assay used by Walsh et al [29] in these same animals. Peripheral sensation, as measured by the tail flick test, showed no discernable differences between age groups in females or males (Figure 7C,D). Nerve conduction studies in these animals following the assays reported here showed that tail sensory nerve conduction was reduced in the 28 and 32-month-old males and females [29].

### Premature death

**Figure 8:**
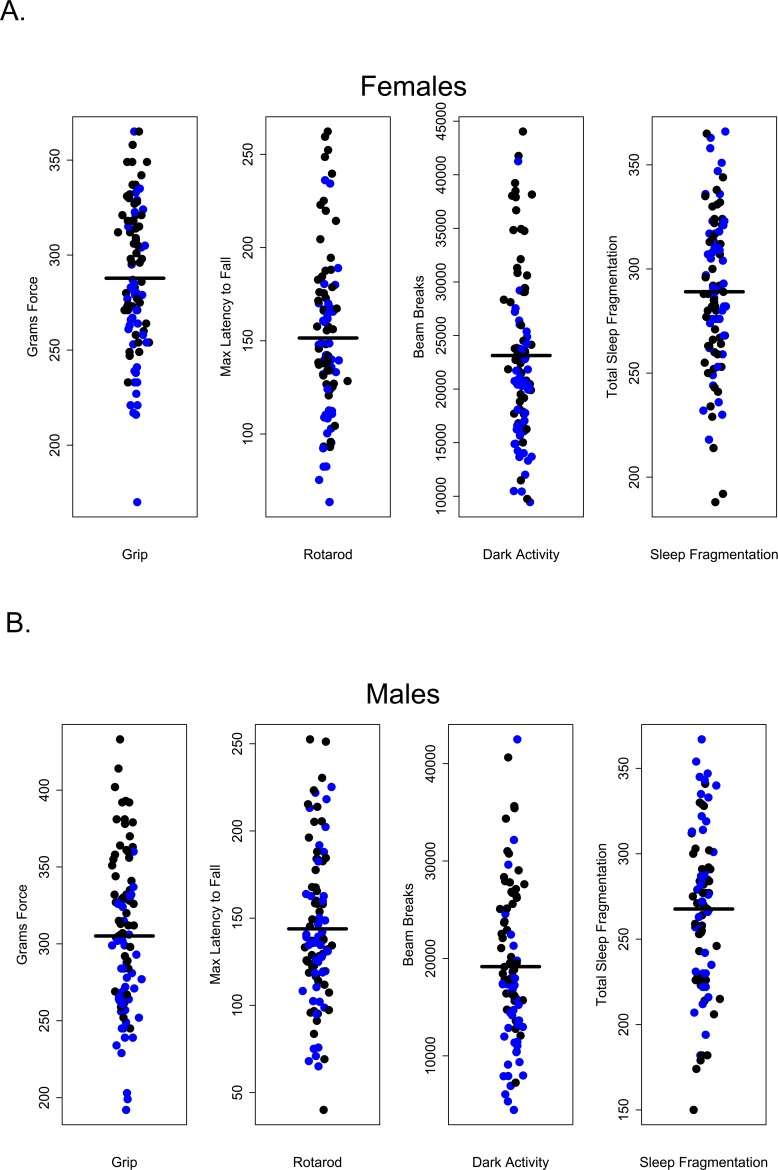
Premature mortality was not correlated with performance in healthspan measures (**A**) Grip strength, rotarod, dark activity and sleep fragmentation were not significantly associated with premature mortality in females, p > 0.99, p = 0.18, p > 0.99, p > 0.99, respectively. (**B**) Grip strength, rotarod, dark activity and sleep fragmentation were not significantly associated with premature mortality in males, p = 0.240, p > 0.99, p > 0.99, p > 0.99, respectively. All tests subjected to Bonferroni corrections for multiple comparisons. Animals suffering premature mortality shown in blue, survivors in black.

Survival in C57BL/6 mice is well documented; 50% survivorship generally occurs about 28 months of age and by 32 months, survivorship is roughly 10% [22]. Very few studies use mice in these two age groups. Nonetheless it is between these two ages that we would expect age-related morbidity and mortality to become manifest, making these age groups clinically relevant. In this study, animals were subject to extensive and invasive measures of health over the course of a 4 to 4.5 month period. Roughly half of all animals (45% of females and 47% of males, respectively), died prematurely over the course of this study. The proportion of males and females within each age group that survived to the end of the study is given in Table. 1; not surprisingly, mortality was greater among 28 and 32-month-old mice in both sexes. After controlling for multiple comparisons, lower percent body fat was associated with premature death but only among females (p = 0.0396); no health measures were significantly associated with premature death in males. This was true even for measures that differed among age groups (Figure 8).

**Table 1:** Premature mortality

	4 months	20 months	28 months	32 months
Female	5% (1)	10% (2)	73% (22)	70% (19)
Male	9% (2)	27% (6)	72% (23)	63% (19)

Percent of each cohort dying prior to the end of the study.

### Correlations between health parameters

**Figure 9:**
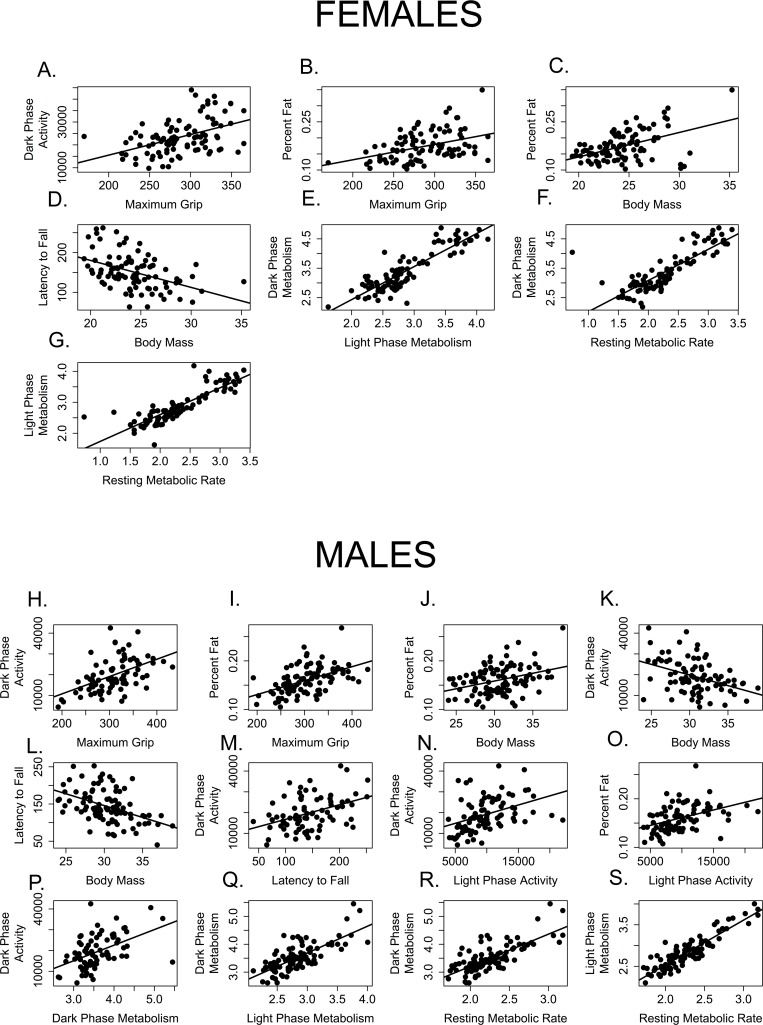
Few measures of healthspan are correlated with one another Measures correlated among female mice include (**A**) grip strength and dark activity (p = 0.0003, R = 0.454); (**B**) grip strength and percent fat (p = 0.0037, R = 0.405); (**C**) percent fat and body mass, (**D**) rotarod performance (latency to fall) and body mass (p = 0.0006, R = −0.443); (**E**) dark and light phase metabolic rate (p < 0.0001, R = 0.880); (**F**) dark phase and resting metabolic rate (p < 0.0001, R = 0.823) and (**G**) light phase and resting metabolic rate (p < 0.0001, R = 0.860). Measures correlated among male mice include (**H**) grip strength and dark activity (p = 0.0003, R = 0.454); (**I**) grip strength and percent fat (p = 0.0037, R = 0.405); **J.** percent fat and body mass; (**K**) dark phase activity and body mass; (**L**) rotarod performance (latency to fall) and body mass (p = 0.0006, R = −0.443); (**M**) rotarod performance and dark phase activity; (**N**) dark and light phase activity; (**O**) percent fat and light phase activity; (**P**) dark phase activity and metabolic rate; (**Q**). dark and light phase metabolic rate (p < 0.0001, R = 0.880); (**R**) dark phase and resting metabolic rate (p < 0.0001, R = 0.823) and (**S**) light phase and resting metabolic rate (p < 0.0001, R = 0.860).

Just as we have assumed that extending lifespan will also extend healthspan, we expect that health in one domain will be correlated with health in others. In other words, we expect measures of healthspan to be maintained or decline together, such that a mouse that shows superior grip strength will also perform better on the rotarod, be more active, etc. Our correlation analysis suggests otherwise, at least for assays used in this cross sectional study. The twelve health measures analyzed here are poorly correlated with one another (Tables 2 and 3, Figure 9). After correcting for multiple comparisons, 18% (12/66) of bivariate correlations among males and only 11% (7/66) bivariate correlations among females were significant (Figure 9). All health measures that were correlated in females were also significantly correlated in males.

**Table 2:** Correlations of female health measures

	Weight	Dark Activity	Light Activity	Maximum Grip	Tail Flick	Max Rotarod	Stride Length	Stance Width	Light $\dot{V}O_{2}$	Dark $\dot{V}O_{2}$	RMR	% Fat
Weight		−0.2395	0.0275	0.0539	−0.0259	**−0.4434**	0.3146	0.2604	0.1076	0.0524	0.1862	**0.4728**
Dark Activity	>0.999		0.1886	**0.4536**	−0.013	0.1385	−0.0383	−0.1324	−0.0036	0.1237	0.055	0.2271
Light Activity	>0.999	>0.999		−0.0803	−0.1081	−0.1279	−0.2693	0.1656	−0.0854	−0.0461	−0.0578	−0.0024
Maximum Grip	>0.999	**0.0003**	>0.999		0.0432	−0.0188	0.1787	−0.117	0.1528	0.227	0.2172	**0.4049**
Tail Flick	>0.999	>0.999	>0.999	>0.999		0.1339	−0.0289	−0.109	0.2437	0.2886	0.2032	0.0001
Max Rotarod	**0.0006**	>0.999	>0.999	>0.999	>0.999		−0.1441	−0.2496	0.0744	0.148	0.0429	−0.0855
Stride Length	0.133	>0.999	0.573	>0.999	>0.999	>0.999		−0.0046	0.1653	0.152	0.2168	0.3023
Stance Width	0.7432	>0.999	>0.999	>0.999	>0.999	>0.999	>0.999		0.1972	0.0921	0.1917	0.0391
Light $\dot{V}O_{2}$	>0.999	>0.999	>0.999	>0.999	>0.999	>0.999	>0.999	>0.999		**0.8795**	**0.8597**	0.2088
Dark $\dot{V}O_{2}$	>0.999	>0.999	>0.999	>0.999	0.3479	>0.999	>0.999	>0.999	**<0.0001**		**0.8229**	0.218
RMR	>0.999	>0.999	>0.999	>0.999	>0.999	>0.999	>0.999	>0.999	**<0.0001**	**<0.0001**		0.2721
% Fat	**0.0001**	>0.999	>0.999	**0.0037**	>0.999	>0.999	0.2019	>0.999	>0.999	>0.999	0.5732	

Values above the diagonal are correlation coefficients, and values below diagonal are p-values. Bold values indicate significant values after Bonferroni correction.

**Table 3:** Correlations of male health measures

	Weight	Dark Activity	Light Activity	Maxi Grip	Tail Flick	Max Rotarod	Stride Length	Stance Width	Light $\dot{V}O_{2}$	Dark $\dot{V}O_{2}$	RMR	% Fat
Weight		**−0.4069**	0.0137	−0.0819	0.02	**−0.4478**	0.1283	−0.0834	−0.2955	−0.3018	−0.2414	**0.3432**
Dark Activity	**0.0074**		**0.4009**	0.**5053**	−0.0095	**0.4091**	−0.1761	0.0435	0.3525	**0.4876**	0.3329	0.293
Light Activity	>0.999	**0.0094**		0.3584	0.0168	−0.0244	−0.2017	−0.1551	0.0558	0.1697	0.0833	**0.4294**
Max Grip	>0.999	**0.0001**	0.0781		0.02	0.2512	0.1875	0.0535	0.2516	0.316	0.2055	**0.504**
Tail Flick	>0.999	>0.999	>0.999	>0.999		−0.0674	−0.0056	−0.102	−0.1186	−0.1642	−0.1038	−0.0593
Max Rotarod	**0.0003**	**0.0132**	>0.999	0.8937	>0.999		−0.08	0.0986	0.1807	0.2737	0.1976	−0.1179
Stride Length	>0.999	>0.999	>0.999	>0.999	>0.999	>0.999		−0.1506	0.1197	0.0413	0.1145	0.0812
Stance Width	>0.999	>0.999	>0.999	>0.999	>0.999	>0.999	>0.999		0.1714	0.1811	0.192	0.0654
Light $\dot{V}O_{2}$	0.1967	0.1021	>0.999	>0.999	>0.999	>0.999	>0.999	>0.999		**0.7389**	**0.8988**	0.159
Dark $\dot{V}O_{2}$	0.1582	**0.0004**	>0.999	0.1418	>0.999	>0.999	>0.999	>0.999	**<0.0001**		**0.7409**	0.1468
RMR	>0.999	0.1914	>0.999	>0.999	>0.999	>0.999	>0.999	>0.999	**<0.0001**	**<0.0001**		0.121
% Fat	**0.0209**	0.4288	**0.0027**	**<0.0001**	>0.999	>0.999	>0.999	>0.999	>0.999	>0.999	>0.999	

Values above the diagonal are correlation coefficients, and values below diagonal are p-values. Bold values indicate significant values after Bonferroni correction.

Predictably, dark and light phase mass-specific metabolism and RMR were all positively correlated with each other for both females (Table 2, Figure 9 E, F, G) and males (Table 3; Figure 9 Q, R, S). Similarly, body mass and rotarod performance were negatively correlated for both males and females, demonstrating that larger animals perform more poorly at this task (Tables 2, 3; Figures 9D, L). Not surprisingly, percent body fat and body mass were positively correlated in both males and females (Table 2, 3; Figures 9C, J). More interestingly, levels of activity during the dark (=active) phase are positively correlated with grip strength in both males and females (Tables 2, 3; Figure 9A, H), and both grip strength and dark phase activity were significantly lower in older mice of both sexes (Figures 2, 7). It is intriguing to note that while body mass and grip strength have no discernable relationship in males or females (p > 0.999 in both cases), there is a significant, positive correlation between grip strength and percent body fat in both males and females (Tables 2, 3; Figures 9B, I). Tail flick latency, rear stance width and rear stride length did not differ between age groups and were not significantly correlated with any other health parameter measured in either sex.

A few significant associations occurred in males only. Body mass and dark phase activity are negatively correlated whilst dark phase activity and rotarod are positively correlated with one another (Table 3, Figure 9K, M), suggesting that males who are more active during the dark phase weigh less and perform better on the rotarod. Conversely, dark phase activity is positively correlated with dark phase mass-specific metabolic rate in males, indicating that males who more active during the dark phase consume more oxygen (Table 3, Figure 9P). Why females failed to show these associations remains an open question.

## DISCUSSION

### Few healthspan measures are correlated with one another

**Figure 10:**
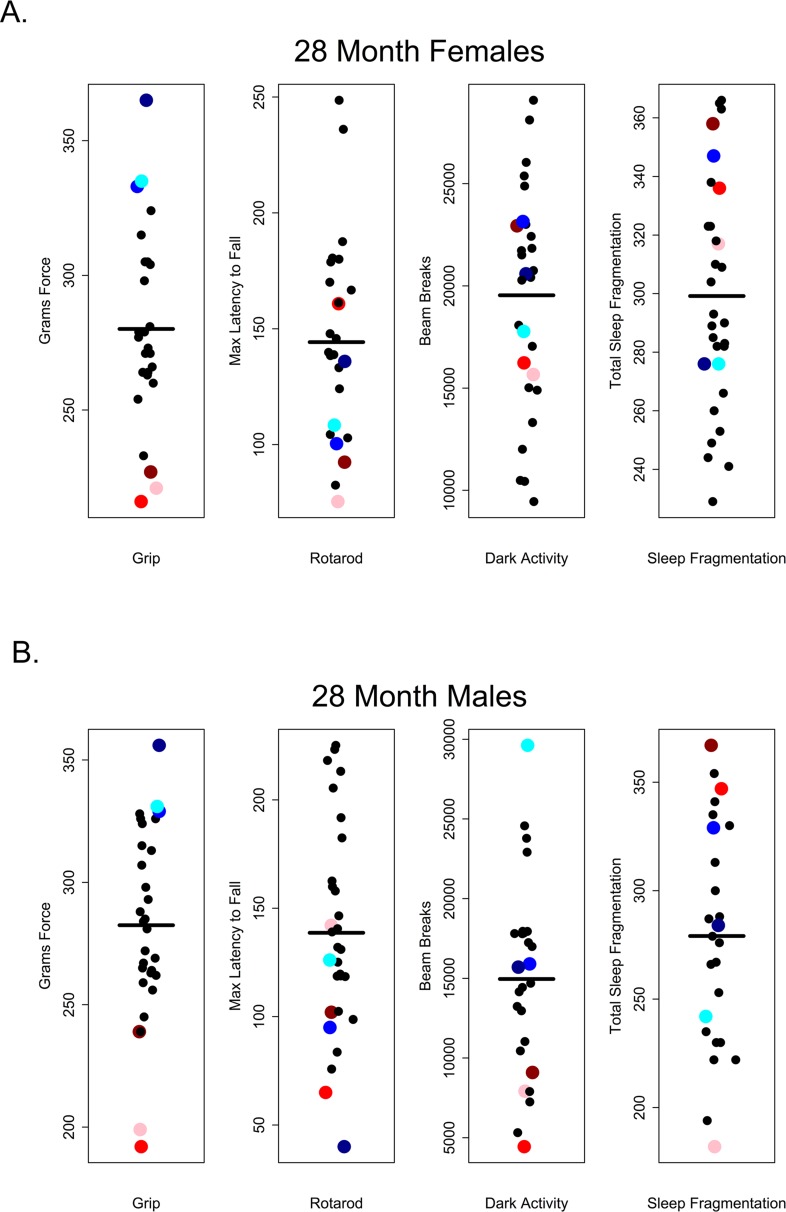
Grip strength does not predict rotarod performance, dark phase activity or sleep fragmentation in 28-month-old mice Visual representation of the relative performance on four healthspan measures by 28-month-old mice. Mice performing best (blue symbols) and worst (red symbols) on the grip strength test, did not perform similarly on other measures of healthspan in females (**A**) and males (**B**). Correlations and p-values are in Tables 2 and 3.

**Figure 11:**
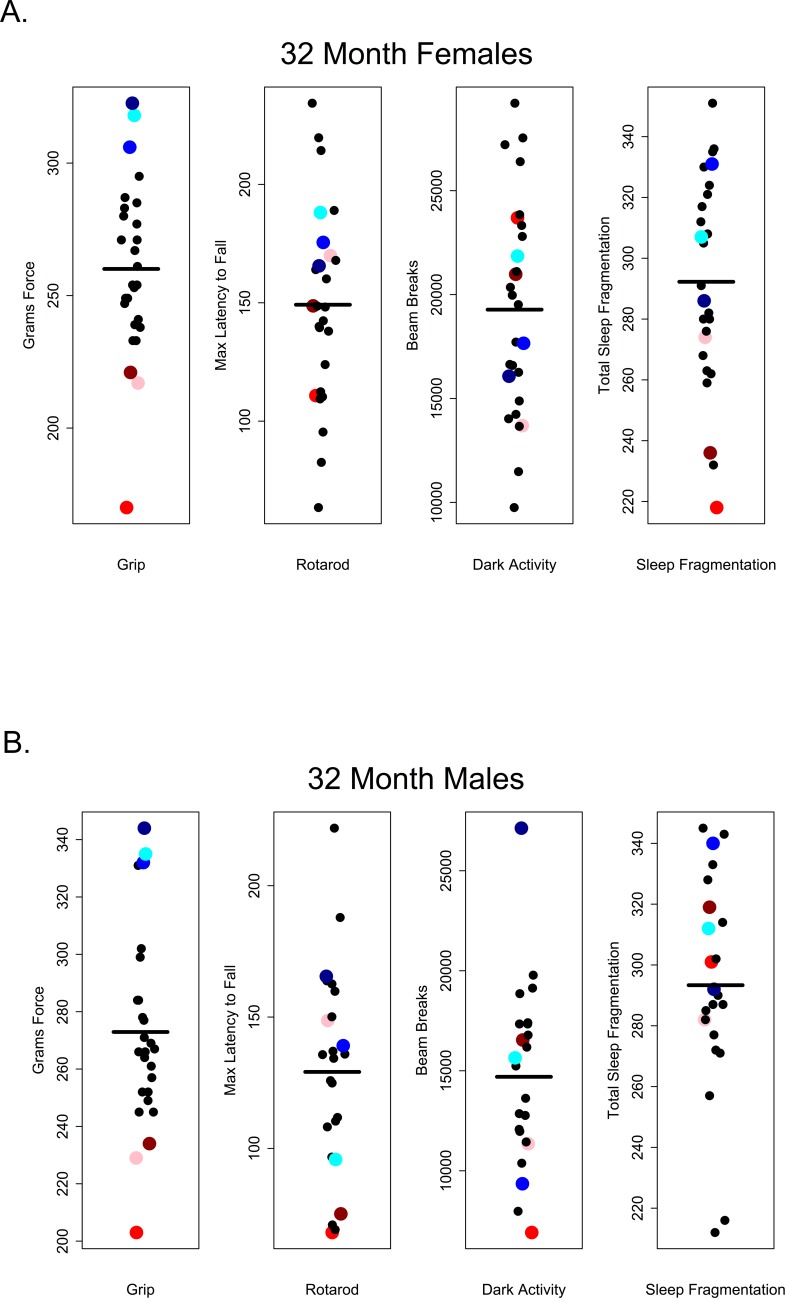
Grip strength does not predict rotarod performance, dark phase activity or sleep fragmentation in 32-month-old mice Visual representation of the relative performance on four healthspan measures by 28-month-old mice. Mice performing best (blue symbols) and worst (red symbols) on the grip strength test, did not perform similarly on other measures of healthspan in females (**A**) and males (**B**). Correlations and p-values are in Tables 2 and 3.

Our results suggest that in older C57BL/6 mice there is very little correlation between performance on one healthspan measure and performance on another as measured in this study. If these assays are robust assessments of age-related changes in health and if age-related declines in function occur globally, we would have expected to see correlations across functional categories of measures. For instance, we might have expected that animals scoring high on tests of grip strength should also perform well other tests; however, there was no evidence of a global decline in health as measured here and very little correlation among tests. Looking at the performance of 28 and 32 month-old mice, those that performed well on the grip strength assay did not necessarily perform well on rotarod, were not consistently more active during the dark phase and did not show less sleep fragmentation. Conversely, poor performance on grip strength was not predictive of performance on other assays (Figures 10 and 11).

Fahlstrom et al [20] looked for correlations among healthspan measures in a cross-sectional study of male C57B/6J mice at 4, 22 and 28 months of age. They examined correlations between measures *within age groups* and found few correlations. The exception was in what they termed ‘exploratory component’ measures, e.g. animals that made few entries in the elevated plus maze also avoided the center of the open field arena. They did find that correlations among measures of the ‘exploratory component’ were maintained in 28-month-old male mice under dietary restriction but not under *ad libitum* feeding; however DR not rescue age-related declines in grip strength, balance or delayed object memory recollection [20]. Falstrom et al did not find significant correlations between body mass and health parameters within age groups. Further studies are needed to understand the relationship between age, body mass and measures of healthspan.

The lack of correlation between healthspan measures observed here suggests several different explanations that are not mutually exclusive. These results may indicate that aging is segmental, affecting organ systems and distinct functional domains differently or at different rates. It is also possible that motivational factors affecting performance on these tests vary between subjects within age groups and may not be consistent from one test to another. Measures that do not require active participation by the mouse may more accurately represent health as opposed to motivation. In this context, it is interesting to note that measures of body mass and percent fat account for 3/7 and 5/12 of the correlations observed in female and male mice respectively.

### Healthspan measures of 28 and 32 month-old mice show few differences

In C57BL/6 mice survivorship declines from roughly 50% to 10% between the ages of 28 and 32 months, so it was somewhat surprising that there were few clear differences in performance on healthspan measures between these two age groups. However, given that this is a cross-sectional study, it is likely that there is a robust survival bias influencing these results. It will be necessary to use longitudinal data with repeated measures of the same individuals to assess whether there are significant changes in these and other measures of healthspan in older mice.

### Healthspan measures were not correlated with premature mortality

Roughly 50% of individuals in both sexes were classified as dying prematurely during this study but only one measure in one sex was of predictive value. Percent body fat, in females only, was associated with premature death after controlling for the effects of age. We found no health parameters in males that were significantly associated with premature death after applying a correction for multiple comparisons. In this study, healthspan measures were taken only once and, within age groups, individual performance often varied widely (e.g. dark phase activity). A better and more statistically powerful strategy is to use multiple measures *longitudinally* in the same individuals and compare changes from baseline in these measures rather than comparing absolute values. Nonetheless, our results suggest the possibility that some measures of healthspan may be uncoupled from lifespan in the mouse, as has previously been shown in *C. elegans* [5]. Survival studies using minimally invasive longitudinal measures of healthspan are needed to discern the extent to which lifespan and individual measures of healthspan are correlated in mice.

### Challenges for the study of healthspan in aging rodents

The results presented here are part of a larger collaborative effort to assess healthspan in aging mice. As part of that effort, we have described a large suite of healthspan measures, discussing their utility and limitations [31]. This study was undertaken in hopes of identifying relatively non-invasive, low-cost measures that would allow researchers to assess whether interventions that extend lifespan also extend health in mice. The only measures of health in which we found significant age-related changes in both sexes were body mass, body composition, grip strength, sleep fragmentation and activity during the 12-hour dark (=active) phase of the 24 hour light cycle. These assays are simple to implement, relatively non-invasive and can be repeated longitudinally so might offer some useful measures of health during aging. In some cases, results from the current study agree with previous studies, but others differ; unfortunately, comparisons between studies are confounded by several factors.

Firstly, because protocols for specific assays vary from lab to lab, it is difficult to compare results across studies. For instance, we measured activity over 24 hours, during one dark and one light phase of the 24-hour light cycle following a minimum of12 hours of habituation. Others, (e.g. [19, 32]) quantify activity over shorter periods ranging from minutes to hours. Likewise, grip strength is measured variously, using a force meter as we do here, by suspending the mouse from a wire rod by its forelimbs and timing how can hang suspended from the rod (e.g. [19, 20]), by allowing a mouse to grasp a wire grid with all four limbs, inverting the grid and timing its fall [14] or by pulling the mouse away from the cage lid and categorizing its grip as sustained, reduced or none [18]. In Table 4, we compare results of this study with three other cross-sectional studies of aging C57BL/6 mice [19, 20, 21]. Grip strength was reduced in older animals of both sexes in all studies, even though the techniques used differed; thus grip strength may provide a robust assessment of neuromuscular function in aging mice. Activity measures varied in both the time of day (light phase vs. dark phase) and the duration of time the animals were monitored (5 minutes to 12 hours). It is not clear that quantifying 5 minutes of spontaneous activity in a lighted arena is measuring the same phenomenon as quantifying activity over a 24 hour light-dark cycle. Not surprisingly, the results were inconsistent between studies, which might be a function of the methods but it could also be a consequence of the sex and/or age groups included in the studies. Similarly, older male mice appear to perform worse at the rotarod task in 3 out 4 studies but females showed no age-related differences in this study.

**Table 4:** Results of three common healthspan measures in four studies of aged C57BL/6 mice

Measure	Assay	Comparison	Ages (months)	Aged Females	Aged Males	Citation
Grip strength	Force gauge	Cross-sectional	4, 20, 28, 32	Lower	Lower	This study
Grip strength	Wire hang	Cross-sectional	3, 8, 15, 30	Lower		[19]
Grip strength	Wire hang	Cross-sectional	4, 22, 28		Lower	[20]
Grip strength	Force gauge	Cross-sectional	3, 20, 26		Lower	[21]
Activity	12 hour, dark phase	Cross-sectional	4, 20, 28, 32	Lower	Lower	This study
Activity	90 min, light phase	Cross-sectional	3, 8, 15, 30	Little difference		[19]
Activity	60 min, light phase	Cross-sectional	4, 22, 28		Lower	[20]
Activity	5 min, light phase	Cross-sectional	3, 20, 26		Lower	[21]
Rotarod	Accelerating 4-40rpm, 300 sec	Cross-sectional	4, 20, 28, 32	No difference	Lower	This study
Rotarod	Accelerating 0-40rpm, 300 sec	Cross-sectional	4, 22, 28		Lower	[20]
Rotarod	Accelerating 4-40rpm, 300 sec	Cross-sectional	3, 20, 26		Lower	[21]

A second factor is that body size, which varies with age, sex and treatment, influences many of the healthspan assays we use (Figure 9). In the current study, male rotarod performance differed among age groups when assessed using analysis of variance (ANOVA); however, there was no effect of age once the effect of body mass was removed (ANCOVA, p = 0.752). Small mice consistently perform better on the rotarod than large mice at all ages. Healthspan measures influenced by body mass are used in many studies of health, frailty, and interventions that affect aging [14, 16, 33]; however, our results suggest these analyses may confound age with age-related changes in body mass or treatment effects on body mass, making these unreliable measures of age-related changes of health in the mouse. For instance, age-matched animals on caloric restriction and long-lived growth hormone mutants may perform better than controls on the rotarod because their neuromuscular function is better preserved, because they are smaller, or both. Similarly, suspension tests may conflate age-related increase in body size with age-related decreases in strength. A study to determine whether age-related changes in body mass and differences in techniques influence the outcomes healthspan assays would prove useful in settling these issues.

Researchers exploring the relationship between health and aging in other model organisms (*Drosophila and Caenorhabditis elegans*) have developed batteries of tests to assess healthspan in many of the domains studied in rodents (Table 5). Measures of activity, motor function, body composition, energy expenditure and cognition have been developed that are broadly comparable to the tests used in rodents. Some measures of healthspan, such as cardiac and immune function, are better developed in rodent models. However, other aspects of healthspan like fecundity and stress resistance are more readily measured in worms and flies (Table 5). It is instructive to compare outcomes across taxa; for example, recent work from the Tissenbaum lab suggests that healthspan may be uncoupled from lifespan extension in long-lived mutants of *C. elegans* [5]. It would be useful to fully develop a schema that crosswalks healthspan measures among model organisms and humans, allowing the results found in one system to be compared with those observed in others. Future work should focus on explicitly assessing the extent to which measures of health and healthspan within functional domains can be translated between model organisms and whether the low correlation between measures of healthspan and extended lifespan is consistent across taxa.

**Table 5:** Availability of healthspan measures in model organisms

Trait	Mice[31]	Flies[34]	Worms[5, 35]
Strength	Grip strength, wire hang	–	–
Motor function	Rotarod, spontaneous activity, Voluntary wheel running,	Vertical climbing, Spontaneous activity	Speed, distance traveled
Endurance	Treadmill,	Negative geotaxis tests	Thrashing
Energy intake	Food consumption	Food consumption	Pharyngeal pumping
Body composition	DXA, QMR, μCT, carcass analysis	QMR	–
Energy expenditure	Indirect calorimetry, doubly labeled water	Indirect calorimetry	Indirect calorimetry
Sleep	Video, EEG, motion detection	Motion detection	–
Fecundity	Litter number, size, and age at last litter	Egg & offspring production, last reproduction	Egg & offspring production, last reproduction
Cardiac function	Echocardiography, blood pressure	Motion detection	–
Immune function	Immune challenge, CD4 & CD8, T-cell function	Immune challenge, antimicrobial peptides	–
Sensory function	Hearing, olfaction, vision, proprioception	Vision, olfaction, proprioception	Chemosensory response
Cognition	Mazes, aversive conditioning	Mazes, aversive conditioning	–
Stress resistance	Cold, toxin resistance	Heat, oxidative stress, starvation, dehydration	Heat, oxidative, osmotic, metal, biotic stress, starvation
Pathology	Standard	Gut homeostasis, lipofuscin and AGEs	Lipofuscin and AGEs

Many measures of health that show no age-related changes in this study, but these results should be interpreted cautiously. There are well-documented strain specific responses to healthspan interventions [36], and so the results reported here may be specific to C57BL/6Nia. Furthermore, because this study is cross-sectional we are not documenting changes in individuals as they age, rather we are describing differences characteristic of mice in different age groups and using cross-sectional studies to draw inferences about longitudinal processes such as aging can be misleading [37]. Nonetheless, the majority of rodent studies are cross-sectional and the work reported here examines commonly used measures of healthspan in the most commonly used rodent strain, from both males and females and at older ages (28 and 32 months) than those generally reported.

To understand the associations between health measures, healthspan and lifespan, a prospective longitudinal study is required such that the same animals are measured using a diverse suite of non-invasive measures of health at regular intervals and exact time of death is known. The limitations of this study and the results of this and other healthspan assessments suggest more work is needed to fully understand how health parameters change throughout the life course of the mouse.

## CONCLUSIONS

Here, we have presented the results of a systematic evaluation of age-related health assays in C57BL/6Nia mice. While some measures showed differed among age groups, these differences were often sex-specific, rarely correlated with one another, and, with one exception, were not predictive of premature death in the animals. Those measures that do stand out, body composition, grip strength and activity during the dark (=active) phase of the 24 hour light cycle are relatively non-invasive and could be standardized so that comparisons across labs, between strains and in longevity-extending interventions are possible. Surprisingly, measures of healthspan were not correlated with one another and performance in one measure was not generally predictive of performance in other measures. The few correlations we did see grouped measures that were functionally related such as our three measures of metabolism. This lack of correlation between measures of healthspan and between measures of health and premature death could be a consequence of the measures used, the cross-sectional nature of this study, the sample sizes or could suggest that aging is segmental and aging organ systems are uncoupled from one another. These results suggest that we have much work ahead to develop robust useful measures of healthspan in the mouse as well as describing the physiological relationship between healthspan and lifespan.

## MATERIALS AND METHODS

### Animals and husbandry

In this cross-sectional study, female and male C57BL/6JNia mice obtained from the NIA colony maintained by Charles River Laboratory (Stone Ridge, NY) at 4, 20, 28 and 32 months of age (females, n = 20, 19, 30, 27; males, n = 22, 22, 32 and 30 respectively) were subjected to a battery of strength and mobility tests and were assessed for body composition and 24 hour energy expenditure at all four ages. Mice were received in groups of 10 animals of a single sex, at approximately 6 week intervals. Each shipment contained 2-3 animals in each age group, allowing for simultaneous testing of animals across ages.

All animals were individually housed (Tek Fresh bedding (Harlan Laboratories)), with food (7912.15 Irradiated Teklad LM Mouse/Rat diet, Harlan Laboratories) and water *ad libitum*. The mice were maintained on a 12/12 Light/Dark cycle at 24°C +/− 1.67 with 30-70% humidity. While at Charles River Laboratories prior to their arrival, the mice were housed with wood shaving bedding; food (sterilized NIH31 diet) and water were available *ad libitum*. Mice were acclimated for two weeks after arrival at UTHSCSA before the tests described here began. The assays reported here took place over a one-month testing period, thus animals that were entered into testing at 32 months of age were 33 months of age at the end of those assays.

The assays reported here were part of a larger suite of measures aimed at determining the trajectory of age-related changes physiological function and health in male and female C57BL/6 mice. The goal was to evaluate health in multiple domains in the same mouse using four cohorts in distinct age groups. In addition to the energetics, strength and mobility assays reported here these mice were subject to tests of neuromuscular, cardiac, visual, renal, immune, bone, and respiratory health over a four and a half month period in these animals (Table 6). Assays ranged from the minimally invasive (e.g. cognitive function and gait measures) to those requiring anesthesia (e.g. cardiac and neuromuscular function) (Table 6). Some studies took place over an extended period and involved repeated sampling; for example, immune function was measured over the course of 4 weeks and involved sampling at multiple time-points. Results from these companion studies have been [29, 38] or will be reported elsewhere. Testing was conducted at two sites. At the Barshop Institute measures of body composition, indirect calorimetry, spontaneous activity over 24 hours, olfactory acuity, rotarod, gait analysis, tail flick, novel object recognition, grip strength, neuromuscular function and echocardiography/Doppler were performed over a period of 8 weeks. Animals were then transferred to the medical school at UTHSCSA, where they were acclimated and underwent further testing. Those assays included tests of visual acuity (optokinetic response), renal function, immune function, bone density (μ-CT, DEXA) and a terminal assay of lung function. By the time the final assay was conducted the animals were 4 to 4.5 months older than at the start of the study. The assays reported here were conducted at the Barshop Institute during the first 4 weeks and, since they were among the least invasive, were the first to be completed.

**Table 6:** Assays performed over the course of this study in order of completion

Assays performed at the Barshop Institute (in order of completion, *reported here)
1st Body Composition (EchoMRI)*
Indirect Calorimetry (Metabolism)*
24 hour Activity Monitoring*
Olfactory Acuity
Rotarod*
Gait Analysis*
Tail Flick*
Novel Object Recognition
Grip Strength*
Neuromuscular Function
2nd Body Composition
Echocardiograph/Doppler

*Transfer to Univ. of Texas Health Science Center Medical School (UTHSCSA)*

**Assays performed at UTHSCSA**
Visual Acuity (Optokinetic Response)
Renal Function
Immune Response
Bone Quality (Micro-computed Tomography & Dual X-ray Absorptiometry)

### Ethics statement

Mice were maintained under barrier conditions at the University of Texas Health Science Center, San Antonio. Care and husbandry of the animals and all procedures for this study were approved by the Institutional Animal Care and Use Committee at the University of Texas Health Science Center at San Antonio. All animals died naturally, were euthanized for health reasons or were euthanized at the end of the study during a terminal assay. Health was closely monitored daily for all animals by project staff; animals that showed evidence of becoming ill were checked more frequently until the condition resolved or the animal was euthanized. Terminally ill animals were euthanized via exposure to CO_2_, followed by cervical dislocation.

### Body composition

Body mass, fat-free mass and fat mass were measured for each mouse using a quantitative nuclear magnetic resonance system (Echo MRI 3-in-1 System, Houston, TX).

### Energetics and spontaneous activity

Oxygen consumption (VO_2_) and carbon dioxide production (VCO_2_) were measured via indirect calorimetry for each mouse using the MARS system from Sables Systems (Las Vegas, NV). Mice were individually housed in a shoebox cage (18.5cm x 8.5cm x 12.5cm) with bedding, food and water (ad libitum) for approximately 48 hours. Each cage was connected to a flow pump, pulling the air from the cage through the flow pump, where it would flow downstream through a humidity meter, carbon dioxide and oxygen analyzer. Gas flow was measured over 24 hours following a minimum 12-hour acclimation period. VO_2_ and VCO_2_ were calculated using the ExpeData software from the MARS system. Resting metabolic rate (RMR) was calculated using the lowest ten measures of oxygen consumption over the 24-hour period. Activity was measured separately, using cages arrayed with infrared beams in the x-y plane (MotorMonitor, Kinder Scientific, Poway, CA). Each mouse was placed in a large, Plexiglas cage (38cm x 20cm x 11.5cm) with corncob bedding, and food and water *ad libitum* for a total of 40 hours. Following acclimation, data from a full 24 hour light-dark cycle used in analysis. The number of beam breaks in the X-Y axis plane was recorded and used as a measure activity. Sleep was assessed using infrared beam breaks following the method validated by Pack et al [39]. In brief, periods of inactivity ≥ 40 seconds were scored as sleep. Sleep fragmentation was measured as the number of discrete sleep bouts per hour sleep during the light (=inactive) phase [24].

### Movement and strength parameters

#### Grip strength

Grip strength was measured by gently pulling the mouse by the tail in a horizontal plane parallel with the base plate of the grip strength meter (Columbus Instruments, Columbus, Ohio). A square mesh grid was used and the animal was allowed to grip the mesh with all four paws prior to being pulled. The animal's grip strength was measured as peak force registered. Each mouse was tested ten times, with the maximum grip strength used in analyses.

#### Tail Flick Test

This assay uses a measure of heat sensitivity in the tail as a proxy for proprioception. Mice were habituated in restraint tubes over a period of 3 consecutive days. On the 4th day the mouse, in its restraint tube, was placed on the tail flick instrument (IITC Tail Flick Analgesia Meter, IITC Life Science, Woodland Hills, CA). A halogen light source at 20% intensity produced heat, which is directed on a 4×6mm area on the mouse's tail for 10 seconds or until the mouse moves/flicks its tail, whichever comes first. The amount of time from exposure to the laser until the mouse flicks its tail (latency) was used in analyses.

#### Gait

The ExerGait (Columbus Instruments, Columbus, Ohio) is a treadmill with a clear belt. The mouse is placed on the belt and a video camera records the animal while it walks on the treadmill from below. The belt speed was adjusted for each mouse until the animal could maintain a consistent walking speed. Most animals walked at speeds between 12-15 cm/s. Gait parameters were interpolated from the video using the Treadscan software (Cleversys Inc, Reston, VA).

#### Rotarod

Mice were tested on an accelerating rotarod (Columbus Instruments, Columbus, Ohio) that started at 4 revolutions per minute (rpm), and increased 0.2 rpm/second. The total time the mouse remained on the rod (including passive rotations) was recorded as latency to fall. Mice performed six trials per day and were trained on rotarod for five days over a consecutive two-week period. On the sixth testing day the animals were assessed using six trials with the maximum latency to fall (in seconds) of those six trials used in analyses.

### Premature death

While this was not survival study, we did record any animals that died prematurely during the 4 to 4.5 month assessment period. Animals that died spontaneously during the study or were euthanized for health reasons were classified as premature deaths.

### Statistical methods

Two-way ANOVA was used to analyze the effects of age group, sex and the interaction of these two factors. Bonferroni correction for multiple comparisons was used in post-hoc testing. Analyses were performed using SPSS (IBM Corp. Released 2013. IBM SPSS Statistics for Windows, Version 22.0. Armonk, NY: IBM Corp). Figures were prepared using sing GraphPad Prism version 6.00 for Windows and Mac OS X, GraphPad Software, San Diego California USA, www.graphpad.com.

To determine the extent to which health measures were correlated with each other we computed Pearson correlations among age groups for twelve healthspan measures. Bonferroni corrections were applied to correct for multiple comparisons.

To determine whether specific measures of health status in the mouse were associated with premature death, we ran a logistic regression to assess the effects of individual health measures on premature death. We controlled for the effects of age by including age as an ordered factor (4 months < 20 months < 28 months < 32 months). This allowed us to ascertain if individual health parameters were correlated with an increased probability of premature death independent of the age. For this analysis, we looked at the 12 health measures used in the correlation analysis above. We used a Bonferroni correction to control for the effects of multiple comparisons. Correlation analyses and logistic regression were completed using the statistical program R (www.r-project.org).
